# Multifractal analysis of maize and soybean DNA

**DOI:** 10.1038/s41598-024-60722-2

**Published:** 2024-05-09

**Authors:** J. P. Correia

**Affiliations:** Departamento de Física, Universidade Federal do Rio Grande do Norte, Natal, RN 59072-970 Brasil

**Keywords:** Biophysics, Genetics, Physics, Biological physics, Statistical physics, thermodynamics and nonlinear dynamics

## Abstract

This paper investigates the complexity of DNA sequences in maize and soybean using the multifractal detrended fluctuation analysis (MF-DFA) method, chaos game representation (CGR), and the complexity-entropy plane approach. The study aims to understand the patterns and structures of these DNA sequences, which can provide insights into their genetic makeup and improve crop yield and quality. The results show that maize and soybean DNA sequences exhibit fractal properties, indicating a complex and self-organizing structure. We observe the persistence trend between sequences of base pairs, which indicates long-range correlations between base pairs. We also identified the stochastic nature of the DNA sequences of both species.

## Introduction

Maize, commonly known as corn and soybean, are two of the most significant crops in global agriculture, each playing a vital role in food production, animal feed, and various industries. They are vital sources of calories, proteins, and essential nutrients, contributing to balanced diets and sustainable agricultural practices^1–5^. Additionally, these crops have significant economic importance, supporting livelihoods and driving agricultural industries such as food processing, animal husbandry, and biofuel production^6,7^.

Maize is one of the oldest and most significant cereal crops in the world, and maize has a lengthy and rich history. Native Americans have been cultivating it in the Americas for a very long time, and it was a staple food for ancient civilizations like the Maya and the Aztecs. Maize arose in Europe for the first time in the late 15th century, and it quickly spread to other parts of the world, such as Asia and Africa^8–10^. Similarly, soybean is a highly significant crop with a fascinating history and widespread cultivation. Native to East Asia, soybeans have been cultivated for centuries and have become a global commodity. Initially grown as a staple crop in East Asian civilizations, soybeans gradually made their way to the Americas and Europe through trade and exploration^11,12^.

Today, these commodities are one of the most widely grown crops in the world. The maize has global production reaching a record high of 1.2 billion tonnes in 2020, according to the Food and Agriculture Organization of the United Nations^13,14^. The United States is the largest producer of maize, followed by China, Brazil, and Argentina. These countries account for over 60% of the world’s maize production. Likewise, the world soybean production in 2020 was 353.5 million tonnes, with Brazil being the largest producer at 135.0 million tonnes^15^, followed by the United States at 96.2 million metric tonnes. Other top producers include Argentina, China, and India^14,16^.

On the other hand, understanding the complexity of these DNA sequences in these plants is crucial for improving crop yield and quality^17,18^. One of the ways to analyze the sequences is to determine the fractal properties^19^. Recently, multifractal analysis has emerged as a powerful tool for characterizing the non-linear dynamics of biological systems, including plant genomes. The multifractal analysis provides a quantitative measure of the scale-invariant properties of a system, which can reveal hidden patterns and correlations in the data^20,21^. Of the various statistical tools available for fractal analysis, we can cite the MF-DFA (Multifractal Detrended Fluctuation Analysis) method, proposed by Kantelhardt^20^, which describes different statistical characteristics of time series on different time scales.

Another essential tool is the CGR (Chaos Game Representation). The CGR is a visualization technique that can be used to represent DNA and protein sequences. It was first proposed by Jeffrey and Sander in 1992^22^. The method is based on the idea of iterative plotting points on a two-dimensional grid, where the position of each point is determined by the sequence of nucleotides or amino acids in the input sequence. The resulting image can reveal patterns and features of the sequence that may not be obvious from the raw data^23,24^.

An interesting tool to analyze data in this work is permutation entropy^25,26^. It is a measure of complexity that quantifies the amount of regularity or predictability in a time series. They have been successfully applied in various fields, including physics, biology, finance, and economics^27–30^. It is based on mapping the time series into a sequence of patterns or ranks, which can be analyzed using metrics such as permutation entropy and complexity-entropy plane. The complexity-entropy plane plots the relationship between the permutation entropy and the complexity of the time series, providing insights into the structure and dynamics of the system under investigation^31^.

Due to the importance of maize and soybean not only for the world economy but also for the planet’s food security, in this study, we investigate the properties of sequences DNA of these commodities, using a multifractal detrended fluctuation analysis (MF-DFA) method, Chaos Game Representation, and plane complexity-entropy to analyze the behaviour scale and determine the fractality of nucleotide sequences. For this, we use the database available on the NCBI website^32^. We define a function to transform the sequence of base pairs $\{A, C, G, T\}$ into a time series. Our results indicate that both species exhibit fractal behaviour along DNA sequences and power law correlations between base pairs. The time series generated by DNA sequences present high persistence and stochastic behaviour, with implies that it has a long-term memory and a tendency to remain close to its past values, while its short-term fluctuations are random.

**Figure 1:**
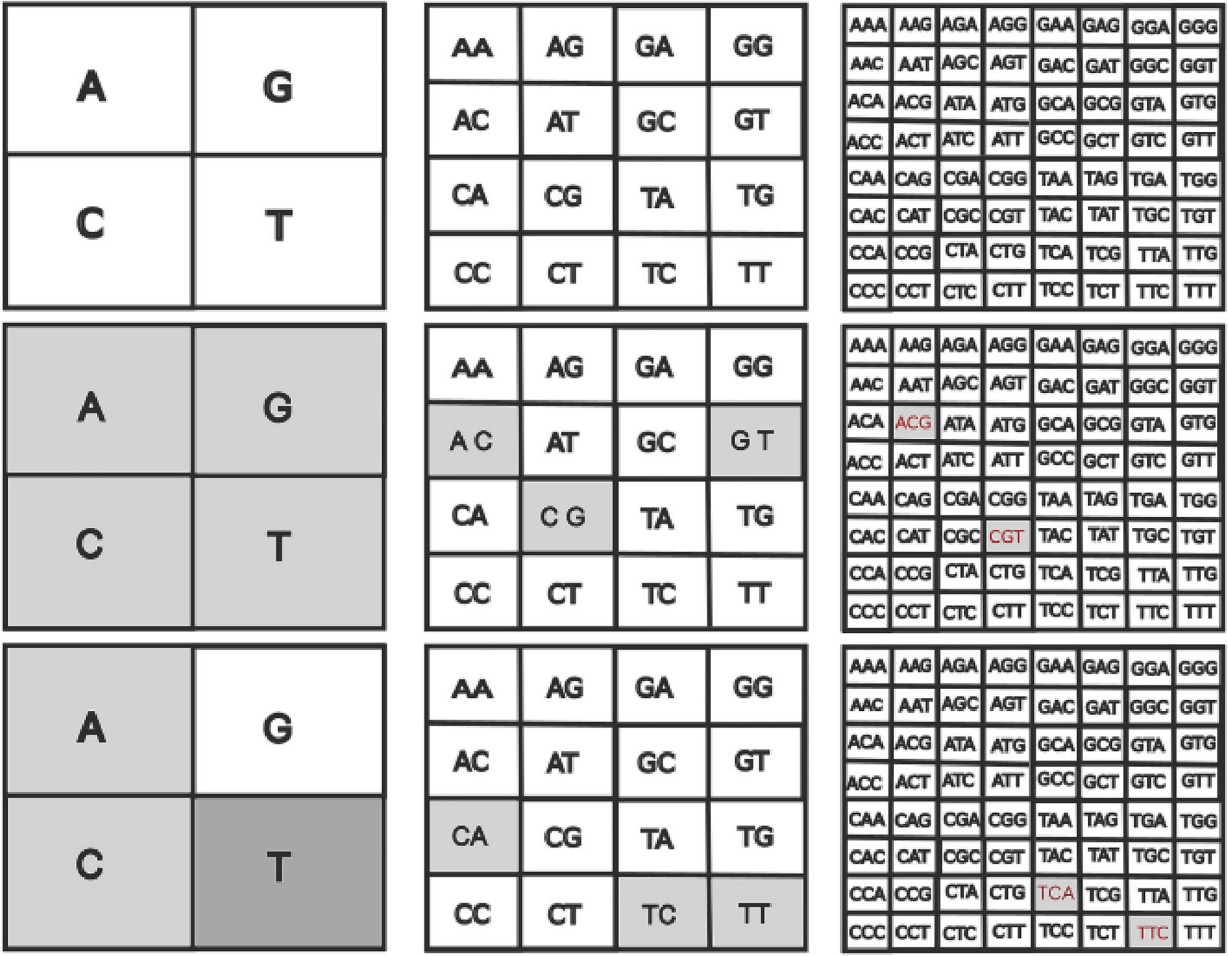
Quadrants in FCGR at different pixelation levels k. In the first line, each quadrant uniquely corresponds to a specific string of length k: $k=1$ (Left column), $k =2$ (Middle column) and $k=3$ (Right column) k = 3 (Top row). In the middle line, we have FCGR of the “ACGT” sequence, with different scales *k*. On the bottom line, FCGR representation of the “TTCA” sequence.

## Theoretical background

### Chaos game representation

The chaos game representation technique is a generalized Markov chain and allows a unique representation of a nucleotide sequence. A mapping rule that transforms a sequence into a two-dimensional picture can reveal fractal structures and has shown promise in recognizing underlying local and global patterns or nucleotide selection bias in gene sequences^23,24^. Mathematically chaos game representation, is described by an iterative system function, where for each new base pair we obtain a set of coordinates (*p*, *q*). The algorithm of this approach follows the following steps^22–24,33^:

1. The nucleotides “A”, “T/U”, “G” and “C” are positioned at the vertices of a square centered at the origin, with coordinates (0, 0). We denote the location of the vertices $V_A= (-1,1)$, $V_C= (-1,-1)$, $V_G = (1,1)$ and $V_T = (1, -1)$ corresponding to the bases *A*, *C*, *G* and *T*, respectively.
2. Given a sequence of base pairs, the first point of the representation is placed at the midpoint between the center of the square and the vertex indicated by the monomer of the first nucleotide.
3. The position of the second point in the representation is obtained by placing it at the midpoint between the position of the first nucleotide and the square of the vertex indicated by the same letter as the second nucleotide.
4. The positions of each subsequent nucleotide are obtained as the midpoint between the position of the previous nucleotide and the vertex corresponding to the current nucleotide. Mathematically, the positions $(p,q)_{i+1}$ are obtained by the recurrence relation:
  1 $$\begin{aligned} (p,q)_{i+1} = \frac{1}{2}((p,q)_i + V_j), \end{aligned}$$
  where $j \in \{A, C, T, G\}$ and we start from the center of the square $(p,q)_0 = (0, 0)$.

In this representation, each point in the CGR corresponds precisely to a subsequence (starting from the first base), and the entire original subsequence of nucleotides up to the current nucleotide can be reconstructed just by knowing the corresponding point in the CGR^34^.

An essential application of the CGR is to assess the abundance of k-mers in a series of nucleotides^34^. A k-mers corresponds to a subsequence of *k* bases. This approach takes advantage of the uneven distribution of subsequences of length *k* $(k = 1, 2, 3, \dots )$ along the nucleotide chain. For example, if we have the DNA sequence “ATCGATCGA” and set k = 3, then the 3-mers would be: ATC, TCG, CGA, GAT, ATC, TCG,CGA. The CGR algorithm generates a square with subquadrants divided by grids where it is possible to represent the frequency of these 3-mers in an image. In Fig. 1 we represent the two-dimensional image generated by the FCGR algorithm. The image is a square with subquadrants where each subquadrant represents a pixel to a given k-mer. For each subquadrant, we associate a gray level that corresponds to the frequency of occurrence of k-mers in the sequence.

Let us consider, for example, the artificial sequence “ACGT”. In this case, each k-mer of length $k=1$ must belong to one of these four quadrants in Fig. 1 middle column on the left. We get one point in each quadrant because we have precisely four different letters in “ACGT”. FCGR counts the occurrence of monomers in each quadrant and assigns a relative grayscale value. Generally, the greater the number of occurrences (the frequency), the darker the quadrant, and vice versa. Therefore, for the string “ACGT”, each corresponding quadrant is represented by the same gray level. For a different sequence, like “TTCA”, we have two points in the T quadrant, one point in the C quadrant, one point in the A quadrant, and no points in the G quadrant. Thus, the gray level of quadrant T is twice as high as that of quadrants C and G, while quadrant A is white, as shown in Fig. 1 lower left line.

The representation $k=3$, in the right column of Fig. 1, corresponds to: For subsequence “ACGT”: ACG, CGT. For subsequence “TTCA”: TTC and TCA. All have the same degree of gray, as they occur with the same frequency in the sequence, and the other representations are blank, as they do not happen in the sequence.

In the same way, we count the frequency of 2-mers in the chains. In the middle row of Fig. 1, we represent the occurrence of the 2-mers for the sequences “ACGT” and “TTCA”. The 2-mers in these sequences have the same shade of gray since they appear with the same frequency, and the other quadrants appear in white since the 2-mers that it represents do not appear. The lower lines on the right of Fig. 1 show the 3-mers representation of the “ACGT” and “TTCA” sequences.

#### Global distance

The Ref.^23^ proposes using the FCGR to determine the dissimilarity between DNA sequences through global distances between sequences on a given scale. For this, we calculate the global distance *d* between two FCGRs based on Pearson’s Weighted Correlation Coefficient, $rw_{p,q}$, using the following equations

2 $$\begin{aligned} nw&= \sum _{i=1}^k p_i . q_i \nonumber \\ \bar{p}w&= \dfrac{\sum _{i=1}^k p_i^2 . q_i}{nw},&\bar{q}w&= \dfrac{\sum _{i=1}^k q_i^2 .p_i}{nw} \nonumber \\ sp&= \dfrac{\sum _{i =1}^k (p_i - \bar{p}w)^2 p_i. q_i}{nw}\nonumber \\ sq&= \dfrac{\sum _{i=1}^k(q_i - \bar{q}w)^2 p_i.q_i}{nw}\nonumber \\ rw_{p,q}&= \dfrac{\sum _{i= 1}^k \frac{p_i-\bar{p}w}{\sqrt{sp}} \frac{q_i- \bar{q}w}{\sqrt{sq}}. p_i .q_i}{nw}, \end{aligned}$$

where *p* and *q* are the coordinates of the quadrants in FCGR, each containing the occurrence of the same *k* oligomeric sequences.

The modification of Pearson’s standard definition consists of weighting the variance with the frequency *nw* to determine the correlation between the two sets of quadrants. The advantage of using this coefficient definition is that the importance of each quadrant is proportional to the frequency of the oligomer it represents. The distance *d* between two DNA sequences is defined by

3 $$\begin{aligned} d = 1 -{rw_{p,q}}, \end{aligned}$$

and has a value between 0 and 2. Values close to zero correspond to exact similarity between sequences and values greater than one would correspond to negative transformation coefficients between sequences. The value of *d* is specific to the resolution of frequency decompositions (FCGR) being detected.

### Time series

The four nitrogenous bases that comprise DNA are represented by the letters $\{A, C, G, T\}$ (adenine, cytosine, guanine, and thymine, respectively). We create a function *f* that maps the four nitrogenous bases that make up the DNA sequence into four distinct values.

In writing, we use the following notation: $f(A) \rightarrow 2$, $f(C) \rightarrow -2$, $f(G) \rightarrow 1$ and $f(T) \rightarrow - 1$. Consequently, we have a sequence of valeus $\{x_k: k=1,2, \dots , N\}$ with $x_k \in \{\pm 1. \pm 2\}$. To build our time series *x*(*t*), we perform a cumulative sum of the values of $x_k$. Each value of the cumulative sum will result in a value that corresponds to a temporal measurement *t*.

A similar definition was used in^34,35^ and aimed to distinguish purines (A and G) from pyrimidines (C, T, U).

### Ordinal patterns

Ordinal pattern methods involve mapping a time series to a sequence of patterns or ranks, where each pattern reflects the order of values in a given window. This mapping enables the study of complex systems by computing various metrics, including permutation entropy and complexity-entropy plane^25,36–38^. In 2002, Bandt and Pompe introduced these methods as a simple, robust, and computationally efficient way to measure complexity in time series data^31^. This measure is defined as the Shannon entropy of a probability distribution associated with ordinal patterns evaluated from partitions of a time series - a process known as the Bandt-Pompe symbolization approach.

Let be $\{x(t): t=1,2, \dots , N\}$ a time series with *N* observations. We divide the series into $n_x = N - (d_x - 1)\tau _x$ non-overlapping partitions, composed of $d_x > 1$ elements and separated by time $\tau _x \ge 1$. For a given $d_x$ and $\tau _x$, we obtain partitions set $w_p = (x_p, x_{p+\tau _x}, \dots , x_{p + (d_x-1)\tau _x})$ where *p* is the index of the partition.

Next, we sort the elements of each partition in ascending order, i.e., for each partition $w_p$, we evaluate the permutation $\pi _p = (r_0, r_1, \dots , r_{d_x+1})$ of the index numbers $(0,1,\dots , d_x-1)$ that sorts the elements of $w_p$ in ascending order. The permutation of the index numbers defined by the inequality $x_{p +r_0} \le x_{p + r_1} \le \dots \le x_{p +r_{dx -1 }}$ e in case of equal values, we maintain the occurrence order of the partition elements. After evaluating the permutation symbols associated with all data partitions, we obtain a symbolic sequence $\{\pi _p\}_{p = 1, \dots , n_x}$. For more details about this method, we recommend the Refs.^25,38,39^

The Ordinal Probability Distribution $\{\rho _{i}({\Pi }_{i})\}_{i=1,...,n_{\pi }}$ is the relative frequency of all possible permutations within the symbolic sequence, given by

$$\begin{aligned} \rho _{i}(\Pi _{i})=\dfrac{\text {number of partitions of type } \Pi _{i} \text { in }\{ \pi _{p}\}}{n_{x}}, \end{aligned}$$

where $\Pi _i$ represents each of $n_{\pi } = d_x!$ different ordinal patterns.

With the ordinal probability distribution, we can calculate the Shannon entropy of permutation

4 $$\begin{aligned} S(P)=-\sum _{i=1}^{n_{\pi }}\rho _{i}(\Pi _{i})\log \rho _{i}(\Pi _{i}). \end{aligned}$$

Entropy, in this context, refers to the degree of disorder or randomness in a time series. Specifically, permutation entropy is a measure of the unpredictability of the order of patterns in a time series such that $S \approx \log n_{\pi }$ indicates randomness and $S \approx 0$ indicates more regular dynamics. Because the maximum value of *S* is $S_{max} = \log n_{\pi }$, we can further define the normalized permutation entropy as

5 $$\begin{aligned} H(P)={\frac{S(P)}{\log n_{\pi }}} \end{aligned}$$

where the value of *H* is restricted to the interval [0, 1].

Another essential measure to characterize a series is complexity. In addition to Bandt and Pompe’s symbolization approach, the complexity-entropy plane is a well-known technique for analyzing time series data^31^. It offers a two-dimensional representation space based on permutation entropy *H* and an intensive statistical complexity measure *C*. This approach, initially created to distinguish between chaotic and stochastic time series, has proven helpful in various situations, including pattern recognition and classification^27,28^.

The statistical complexity measure used in this method was inspired by Lopez Ruiz’s work^40^ and is defined by Jensen-Shannon divergence between the ordinal distribution $P = {\rho _i(\Pi _i)}_{i=1,...,n_{\pi }}$ and the uniform distribution $U = \{1/n_{\pi }\}_{i=1,...,n_{\pi }}$. Mathematically, we can write this complexity as

6 $$\begin{aligned} C(P)={\frac{D(P,U)H(P)}{D^{\textrm{max}}}}, \end{aligned}$$

where

$$D(P,U)=S[(P+U)/2]-{\frac{1}{2}}S(P)-{\frac{1}{2}}S(U),$$

is the Jensen-Shannon divergence and $D^{\textrm{max}}$ is the normalization constant given by

$$D^{\textrm{max}}=-\frac{1}{2}\left( \frac{n_{\pi }!+1}{n_{\pi }!}\log (n_{\pi }!+1)-2\log (2n_{\pi }!)+\log n_{\pi }!\right) .$$

The existence of nontrivial structures is quantified by complexity. The statistical complexity $C = 0$ in both the extremes of order (when only one permutation symbol happens) and disorder (when all permutations are equally likely to occur), in contrast to the permutation entropy, which is non-zero. The value of C measures structural complexity and conveys extra details that the value of H does not. Furthermore, there are a variety of alternative values for C for a given value of H, making C a nontrivial function of H. A more detailed discussion of the meaning of C complexity can be found in Ref.^40^.

### Multifractal dentendred flutuation analysis

Assume that $\{x(t): t = 1,2, \dots N\}$ is a time series with *N* data points. The Multifractal detrended fluctuations analysis procedure consists of the following steps^20^:

1. We determine the profile
  7 $$\begin{aligned} { Y(i) = \sum _{t=1}^i(x(t) - \langle x (t) \rangle ), }\text { for }i = 1,2, \dots N \end{aligned}$$
  where $\langle x(t) \rangle$ is the average of the time series.
2. The profile *Y*(*i*) is divided into $N_s= \text {int}(N/s)$ non-overlapping segments of equal length *s*. Since *N* will not always be a multiple of *s*, a final part of the profile may be left over. To avoid discarding this part of the series, the same procedure is repeated starting from the end. So we will get $2 N_s$ segments.
3. Calculate the local variance for each of the $2N_s$ segments by least squares fit
  8 $$\begin{aligned} F^2(v, s) = \frac{1}{s} \sum _{i=1}^s \{ Y[(v-1)s + i] - y_v(i)\}^2 \end{aligned}$$
  for each segment *v*, $v = 1,2, \dots , N_s$, and
  9 $$\begin{aligned} F^2(v,s) = \frac{1}{s} \sum _{i=1}^N \{ Y[N- (v-N_s)s + i] - y_v(i)\}^2 \end{aligned}$$
  for each segment $v = N_s +1, N_s+2, \dots , 2N_s$. Here $y_v(i)$ is the fit polynomial in the *i* segment and is chosen based on the time series trend. We can use polynomials of different orders in the fitting process so that we will have polynomials of linear (DFA1), quadratic (DFA2), cubic (DFA3), and higher orders.
4. So far, we have obtained *F*(*v*, *s*) which is the variance of each segment *v* of size *s* with an arbitrary polynomial. We define the $q-$th order of the fluctuation function by averaging all $2N_s$ segments
  10 $$\begin{aligned} F_q(s) = \left\{ \frac{1}{2N_s} \sum _{v=1}^{2N_s} [F^2(v, s)]^{q/2} \right\} ^{1/q}. \end{aligned}$$
  When $q=2$, we return the default DFA technique. For different values of *q*, we are interested in how the fluctuation function $F_q(s)$ varies on each length scale *s*. We repeat steps 2 through 4, varying *s*,
5. If there is a long-range power law correlation in the series $x_k$, $F_q(s)$ increases for large values of *s*, mimicking a power law
  11 $$\begin{aligned} F_q(s) \sim s^{h(q)}, \end{aligned}$$
  where *h*(*q*) is the generalized Hurst exponent.

A time series is monofractal if the Hurst exponent *H* remains constant regardless of the value of *q*. On the other hand, if *h*(*q*) varies with *q*, the time series is multifractal. The spectrum of *h*(*q*) is determined by the slopes of the $F_q(s)$ vs. *s* graph for different q values^20,21^. The variations in *h*(*q*) are examined to assess the impact of scale fluctuations. The difference between the asymptotic values of *h*(*q*), denoted as $\Delta h(q)= h_{q_{min}}-h_{q_{max}}$, is computed to measure the departure from monofractal behavior. The parameter $\Delta h(q) =0$ in monofractal series. The magnitude of $\Delta h(q)$ indicates the multifractality and dynamics complexity level in the time series. See References for a more detailed explanation and calculation of the generalized Hurst exponent^41^.

The MF-DFA technique is unsuitable for strongly anti-correlated series where h(q) approaches zero, as it only calculates positive generalized Hurst exponents. In order to address this issue, a modified MF-DFA approach has been recommended. This modification, represented by a double sum substitution in Eq. (7), provides a more appropriate method for analyzing such data^20^

12 $$\begin{aligned} \tilde{Y}= \sum _{k=1}^i [Y(k) - \langle Y \rangle ]. \end{aligned}$$

Following the MF-DFA procedure as described above, we obtain generalized fluctuation functions $\tilde{F}_q(s)$ described by a scaling law as in Eq. (11), but with higher exponents $\tilde{h}(q) = h(q) + 1$

13 $$\begin{aligned} \tilde{F}_q(s) \sim s^{\tilde{h}(q)} = s^{h(q)+1} \end{aligned}$$

Thus, the scaling behavior can be accurately determined even if *h*(*q*) is less than zero for some values of *q*. The multifractal scale exponent $\tau (q)$ of the form can be used to understand the dependency on *q* in the multifractal situation

14 $$\begin{aligned} \tau (q) = q h(q) - 1, \end{aligned}$$

which depends on the generalized Hurst exponent *h*(*q*). The properties of multifractality are more robust as the nonlinear relationship between $\tau$ and *h*(*q*) is more potent.

The multifractal spectrum $(\alpha , f(\alpha ))$, which is related to the multifractal scale spectrum $\tau (q)$ through a first-order Legendre transformation^42,43^, is another approach to represent the multifractal of a time series. If $\tau (q)$ is sufficiently smooth, the singularity’s strength, $\alpha$, is given by

15 $$\begin{aligned} \alpha =\frac{d \tau (q)}{dq} = h(q) +q h'(q), \end{aligned}$$

from which the singularity spectrum $f(\alpha )$ can be constructed

16 $$\begin{aligned} f(\alpha ) = q\alpha - \tau (q). \end{aligned}$$

The graph of $f(\alpha ) \text { vs } \alpha$, also known as the multifractal spectrum or spectrum of singularities, reflects the properties of the profile of *h*(*q*). The exponent $\alpha$ reveals the differences in scale exponents, and the magnitude of the singularity force $\alpha$ is higher for time series with stronger multifractality centered on the prominent scale *h*. The function $f(\alpha )$ reaches its maximum value when $q=0$, with $\max f(\alpha ) = 1$. In a monofractal series, where $\alpha = \tau '(q) = H$, the sets representing $f(\alpha )$ collapse to a single point.

We also define the symmetry parameter B given by

17 $$\begin{aligned} B = \frac{\alpha _{max} - \alpha _0}{\alpha _0 - \alpha _{min}} \ . \end{aligned}$$

The spectrum is symmetric if $B=1$. Subsets exhibiting minor fluctuations generally have a more pronounced impact on the multifractal spectrum when $B>1$, suggesting a directly symmetric spectrum. Conversely, if $B<1$, the multifractal spectrum skews toward the left, with the larger fluctuations tending to exert a greater influence on it. See References for a thorough evaluation of the generalized Hurst coefficients’ significance and interpretation^20,41,44^.

**Figure 2:**
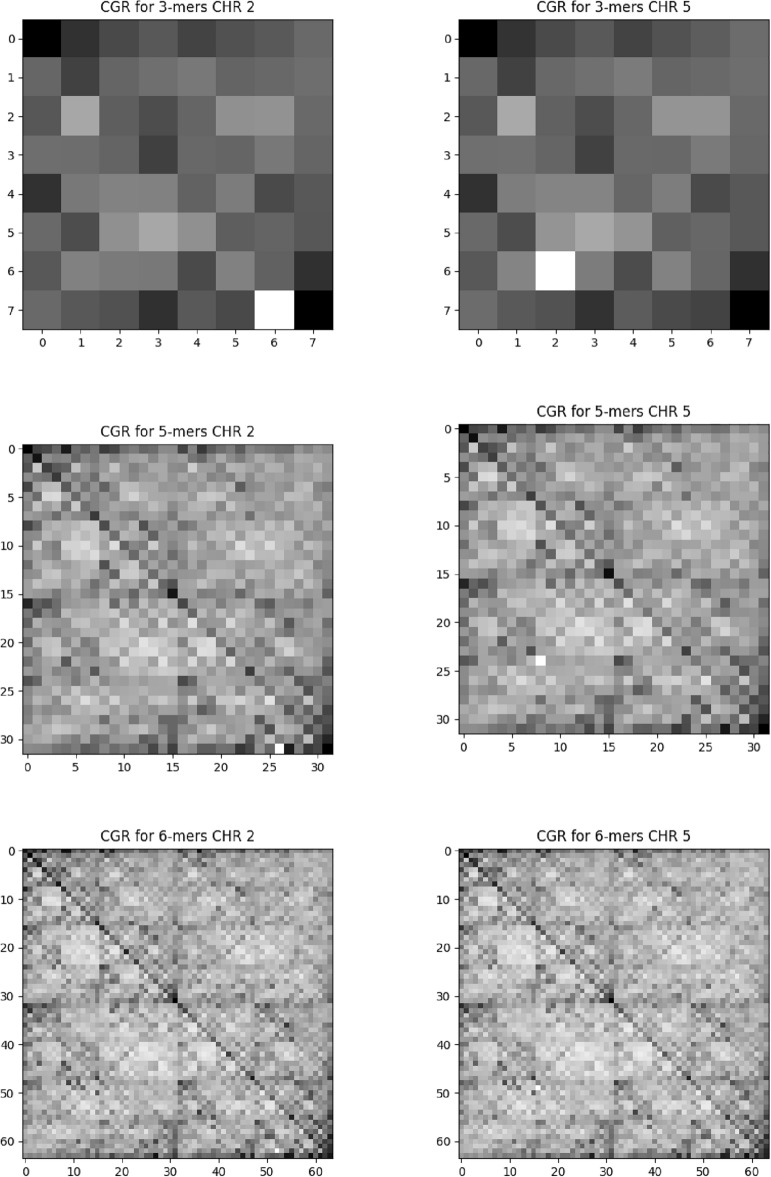
FCGR for randomly chosen maize chromosomes. The first column indicates the results for chromosome 2 and the second for chromosome 5. Each row shows different scales for various *k* scales. Top: $k=3$, middle: $k=5$ and bottom: $k =6$. All maize chromosomes exhibit similar FCGR behavior.

**Figure 3:**
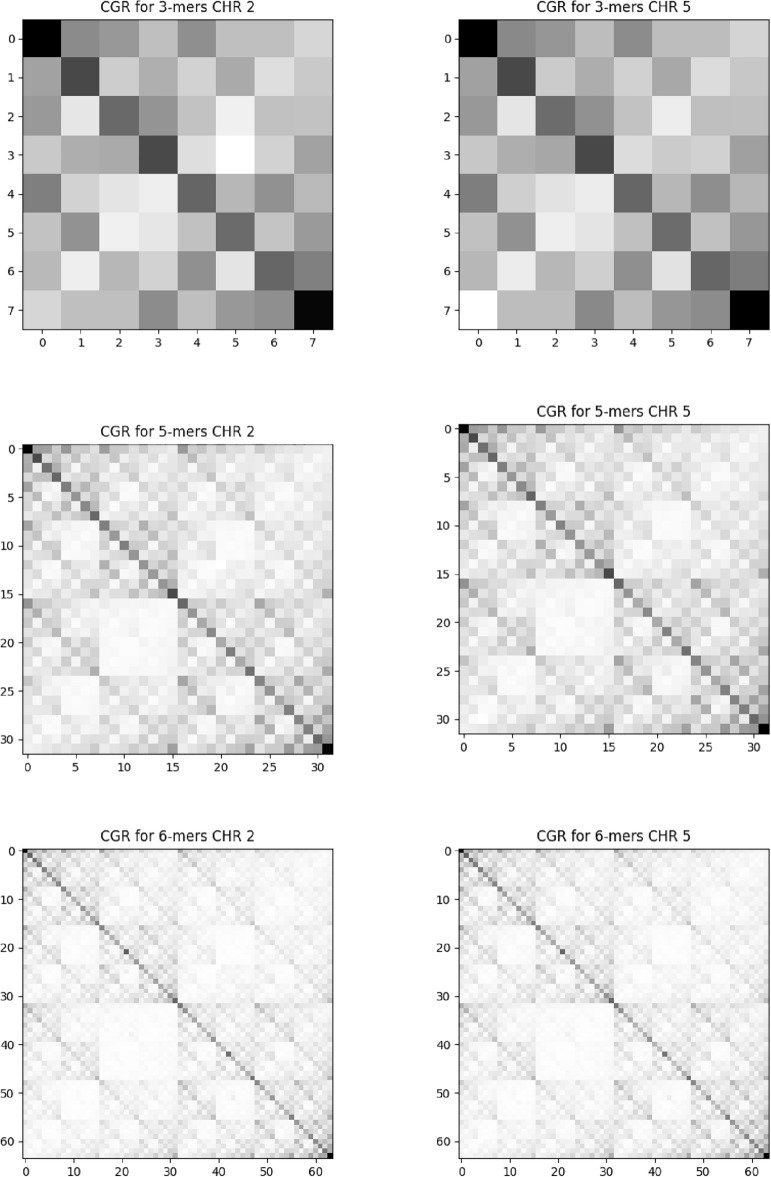
FCGR for soybean chromosomes. The first column indicates the results for chromosome 2 and the second for chromosome 5. Each row shows different scales for various *k* scales. Top: $k=3$, middle: $k=5$ and bottom: $k =6$. All soybean chromosomes exhibit similar FCGR behavior.

**Figure 4:**
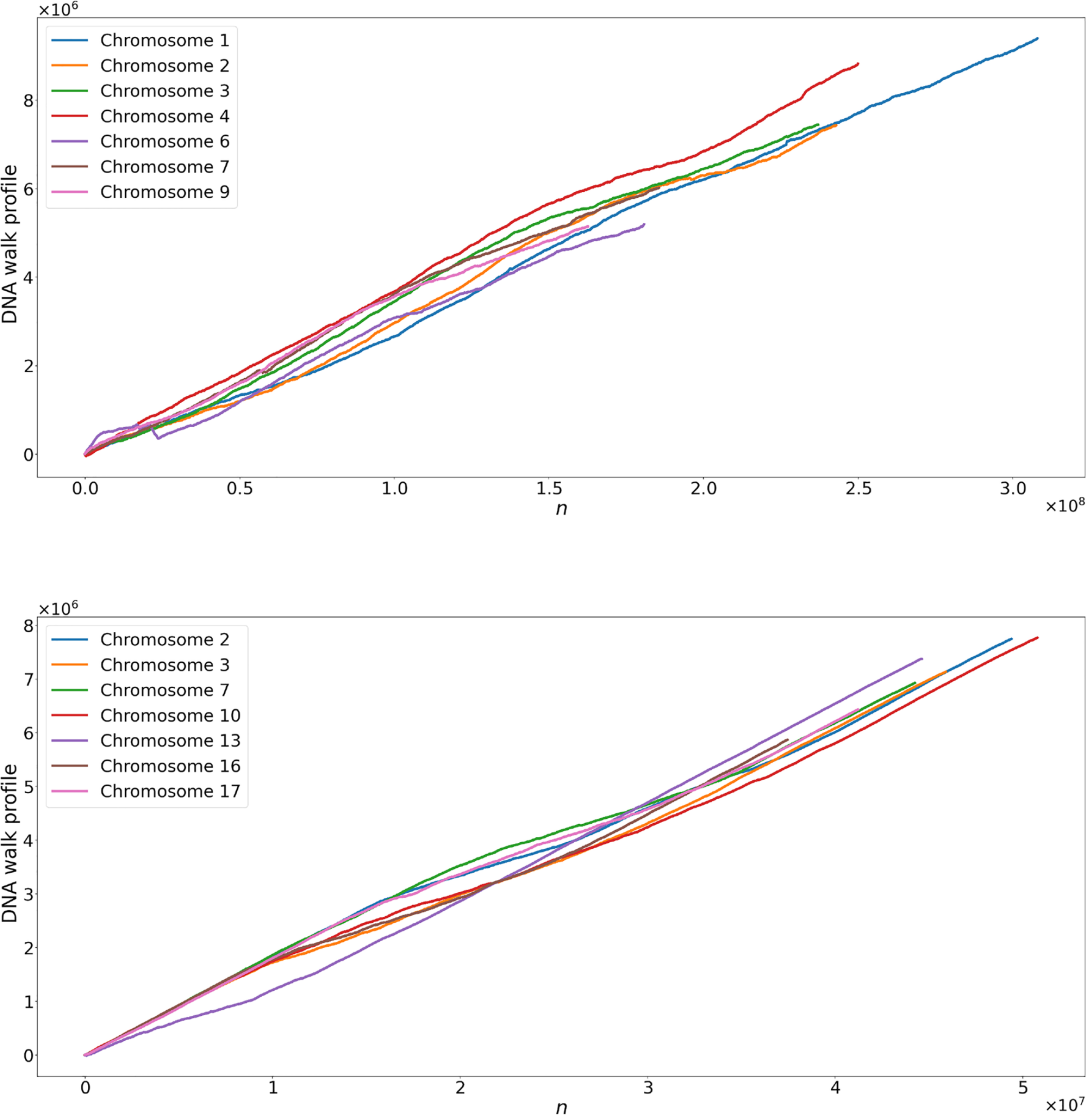
Time series representation of chromosomes maize (top) and soybean (bottom), as described in “Time series” section. Note that all walks tend to go to higher values, meaning a high concentration of bases A and G.

**Figure 5:**
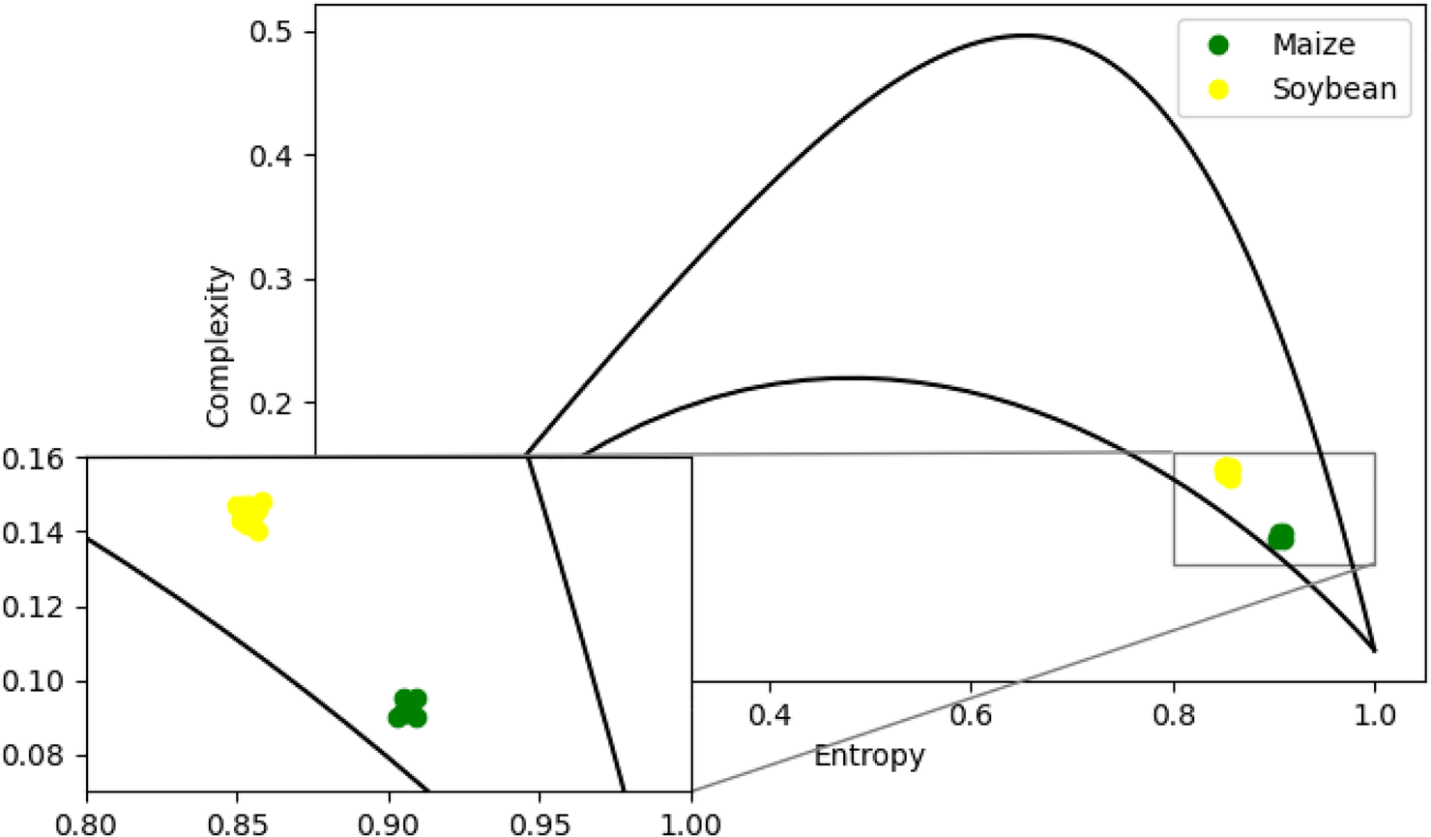
Plane complexity-entropy for maize and soybean chromosomes. Continuous lines represent minimum $C_{min}$ and maximum $C_{max}$ complexities. We zoomed in the region to better visualize the points.

## Results and discutions

Maize and soybean nucleotide sequences are available from the National Center for Biotechnology Information-NCBI^32^. We used the complete sequences of the 10 chromosomes that make up maize and 20 chromosomes that make up soybean to apply the analysis tools.

**Table 1:** Distance matrix *d* between maize chromosomes (horizontal) and soybean chromosomes (vertical), with scale $k=3$.

Soybean	Maize									
	01	02	03	04	05	06	07	08	09	10
01	0.143	0.134	0.139	0.117	0.136	0.131	0.123	0.138	0.136	0.137
02	0.162	0.152	0.159	0.145	0.156	0.149	0.141	0.157	0.155	0.156
03	0.160	0.150	0.156	0.133	0.153	0.147	0.139	0.155	0.153	0.154
04	0.145	0.136	0.141	0.119	0.138	0.133	0.125	0.141	0.139	0.140
05	0.165	0.146	0.152	0.129	0.148	0.142	0.134	0.150	0.148	0.149
06	0.165	0.155	0.191	0.138	0.157	0.151	0.143	0.159	0.158	0.158
07	0.163	0.152	0.158	0.135	0.155	0.149	0.141	0.158	0.155	0.156
08	0.172	0.161	0.168	0.144	0.165	0.158	0.150	0.166	0.165	0.166
09	0.161	0.150	0.157	0.134	0.153	0.148	0.139	0.156	0.153	0.154
10	0.159	0.149	0.155	0.132	0.152	0.146	0.138	0.154	0.152	0.153
11	0.168	0.157	0.164	0.140	0.160	0.154	0.146	0.162	0.160	0.161
12	0.159	0.149	0.155	0.132	0.152	0.146	0.138	0.154	0.152	0.153
13	0.186	0.175	0.182	0.158	0.179	0.172	0.164	0.181	0.178	0.179
14	0.148	0.138	0.144	0.122	0.141	0.135	0.127	0.143	0.141	0.142
15	0.156	0.146	0.152	0.129	0.149	0.143	0.135	0.151	0.149	0.150
16	0.161	0.151	0.157	0.134	0.154	0.148	0.140	0.156	0.154	0.155
17	0.168	0.158	0.164	0.141	0.161	0.155	0.147	0.163	0.161	0.162
18	0.156	0.147	0.153	0.130	0.149	0.143	0.136	0.152	0.149	0.150
19	0.149	0.140	0.146	0.123	0.142	0.136	0.129	0.145	0.142	0.143
20	0.148	0.139	0.145	0.123	0.141	0.136	0.128	0.144	0.142	0.143

**Table 2:** We show the main statistical characteristics of the time series generated by the sequences of base pairs of maize and soybean chromosomes. The first, second, third, fourth, fifth, and sixth columns indicate the chromosome, size of each sample, maximum and minimum values, and the samples’ mean and variance, respectively. We also present the main fractal measures: The seventh column contains the Hurst exponent *H*. The eighth, ninth, and tenth columns are, respectively, variations of $\Delta h = h_{max} - h_{min}$, $\Delta \alpha = \alpha _{max} - \alpha _{min}$ and symmetry parameter *B*.

Maize									
CHR	N	Min	Max	Mean	Variance	H	$\Delta h$	$\Delta \alpha$	B
01	308, 009, 078	$-702$	9, 392, 918	4, 638, 136	7, 953, 513, 754, 802	0.99	0.17	0.26	1.60
02	242, 968, 907	$-189$	7, 433, 507	3, 734, 564	5, 240, 958, 583, 626	0.99	0.66	0.77	8.62
03	237, 196, 652	$-528$	7, 447, 127	3, 914, 916	5, 173, 791, 919, 150	0.99	0.30	0.43	4.37
04	249, 980, 224	$-49,644$	8, 819, 644	4, 492, 570	6, 253, 898, 379, 091	0.99	0.28	0.44	0.47
05	226, 171, 974	$-53,003$	7, 192, 730	3, 591, 582	4, 929, 352, 632, 979	0.98	0.27	0.40	0.48
06	180, 854, 649	$-265$	5, 191, 944	2, 613, 884	2, 483, 349, 000, 349	0.90	0.68	0.86	0.79
07	185, 614, 793	$-461$	6, 017, 835	3, 153, 221	3, 318, 905, 967, 141	0.98	0.43	0.55	5.11
08	182, 214, 470	$-191$	5, 781, 262	3, 033, 165	2, 904, 746, 990, 333	0.98	0.39	0.50	1.77
09	162, 713, 747	$-509$	5, 145, 068	2, 733, 723	2, 404, 245, 834, 613	0.99	0.24	0.36	2.60
10	152, 314, 425	$-2,317$	4, 668, 762	2, 491, 098	2, 001, 048, 999, 103	0.98	0.27	0.38	2.17
Soybean									
01	56, 828, 858	$-4$	8, 305, 962	4, 040, 059	5, 119, 905, 865, 689	0.99	0.63	0.75	36.5
02	49, 417, 934	$-222$	7, 746, 402	3, 898, 106	4, 412, 384, 626, 525	0.99	0.10	0.17	7.5
03	45, 918, 609	$-76$	7, 136, 847	3, 462, 785	3, 843, 596, 419, 558	0.99	0.15	0.23	6.67
04	50, 654, 792	$-55$	7, 357, 721	3, 712, 575	3, 869, 275, 038, 805	0.99	0.16	0.26	12
05	41, 376, 929	$-129$	6, 233, 101	3, 028, 737	2, 826, 337, 391, 010	0.99	0.47	0.59	58
06	49, 072, 608	$-66$	7, 646, 716	3, 937, 325	4, 364, 651, 515, 765	0.99	0.72	0.83	26.6
07	44, 263, 258	$-61$	6, 928, 747	3, 588, 473	3, 593, 563, 463, 972	0.99	0.24	0.35	17
08	46, 810, 571	$-228$	7, 511, 690	3, 869, 876	4, 259, 301, 645, 439	0.99	0.12	0.19	85
09	47, 989, 247	$-327$	7, 330, 542	3, 609, 412	3, 936, 218, 669, 843	0.99	0.87	0.98	31.7
10	50, 795, 466	$-68$	7, 769, 173	3, 794, 438	4, 448, 152, 110, 356	0.99	0.22	0.32	15
11	38, 938, 890	$-323$	6, 065, 233	3, 140, 219	2, 896, 363, 324, 786	0.95	0.10	0.12	13
12	40, 805, 084	$-282$	6, 105, 447	3, 137, 356	2, 737, 977, 759, 860	0.99	0.39	0.52	25
13	44, 628, 233	$-8,622$	7, 375, 225	3, 414, 071	4, 849, 537, 217, 069	0.99	0.15	0.20	9
14	48, 925, 937	$-10$	7, 373, 518	3, 776, 555	4, 024, 438, 759, 515	0.99	0.09	0.15	13
15	50, 694, 678	$-178$	7, 741, 446	4, 049, 917	4, 474, 764, 527, 700	0.99	0.55	0.66	21
16	37, 472, 724	$-273$	5, 868, 029	2, 880, 534	2, 499, 544, 854, 176	0.99	0.14	0.21	4.25
17	41, 228, 219	$-225$	6, 432, 032	3, 308, 281	3, 123, 754, 836, 447	0.99	0.27	0.37	36
18	56, 808, 287	$-71$	8, 690, 214	4, 344, 757	5, 472, 052, 989, 922	0.99	0.33	0.41	19.5
19	50, 139, 364	$-1$	7, 403, 584	3, 536, 675	3, 992, 740, 357, 008	0.99	0.35	0.46	14.3
20	47, 358, 722	$-187$	6, 994, 429	3, 305, 208	3, 674, 593, 366, 604	0.99	0.13	0.17	7.5

### Chaos game representation

We obtained chaos game representations for all 30 chromosomes with different scales *k*. We use the code available in^33^. This representation allows the visualization of repetition patterns in nucleotide sequences. This approach allows us to visualize geometric patterns like parallel lines, squares, rectangles, and triangles. The abundance of nucleotide sequences in the image is reflected through the degree of gray so that the more abundant the *k*, the darker the quadrant that represents it. The CGR image can reveal the overall base composition of the DNA sequence. Different regions of the image correspond to different nucleotide frequencies.

In Fig. 2, we present the frequency of $3-$mers, $5-$mers, and $6-$ mers for the randomly chosen chromosomes $2 \text { and } 5$ for maize. These results correspond to the degree of pixelation $k = 3, 5 \text { and } 6$, respectively. At these degrees of pixelation, all possible combinations of nucleotide sequences are displayed. In Fig. 3, we present the results with the same scales $k = 3,5 \text { and } 6$ for the soybean chromosomes 2 and 5. The other chromosomes present patterns similar to those presented. Visually, the images generated by the soybean sequences appear to have a more explicit fractal behavior, with better-defined geometric patterns.

By generating CGR for all 30 chromosomes using various scales, we identified a range of fractal shapes, including parallel lines, squares, rectangles, and intricate fractal structures. This discovery highlights the underlying principles that govern the arrangement of nucleotides and opens up new ways for understanding the functional and evolutionary aspects of the genome. We can see that the distribution of degrees of gray has a behavior that is not random for both species.

When a Chaos Game Representation (CGR) image displays global patterns of squares and parallel lines, it suggests the presence of specific structural elements or motifs within the DNA sequence. The squares observed in the CGR image indicate regions of the sequence that exhibit repetitive patterns. These squares represent areas where specific nucleotide sequences or structural elements occur repeatedly. Moreover, the presence of parallel lines in the CGR image indicates the presence of periodic or alternating patterns within the DNA sequence. These lines can signify regions where the DNA sequence exhibits a periodicity or a repeated pattern of nucleotides or base compositions.

We calculated the similarity between the chromosomes using Eq. (3) for pixelation level $k=3$. See Table 1. All chromosomes are highly similar, with $0.120< d < 0.190$. It means that all chromosomes are similar, and the distribution of trimers in chromosome sequences is related. In this sense, the species of maize and soybean are very similar.

### Time series and ordinal patterns

This construction step of time series from chromosome sequences is essential for applying the MF-DFA and ordinal patterns methods. We use the *f* mapping rule defined in the “Time series” section. We reinforce that, as different mapping rules can be made to transform a sequence of symbols (DNA sequence) into a time series, we can obtain different fractal parameters that characterize the data. However, as we apply the same rule to both species, we can obtain important information by comparing the obtained fractal parameters. The main statistical characteristics of the resulting time series are shown in Table 2 and the time series representations, for some chromosomes chosen randomly, are shown in Fig. 4 for both species. We can observe in the graphs of the walks that positive values tend to appear, indicating the concentration of A and G in the nucleotide sequences, as defined by our mapping rule.

We calculated entropy *H* and complexity *C* time series generated for all maize and soybean chromosomes. We divide the time series into $n_x$ partitions of sizes $d_x = 3$ and $\tau _x = 1$. We use the Ordpy library introduced by^38^ and available in^45^.

We plot the values obtained in the complexity-entropy plane for each chromosome; see Fig. 5. Entropy values for soybean are around $H \approx 0.855$ and complexity $C \approx 0.145$. For maize, $H\approx 0.906$ and $C \approx 0.091$. Both series have high entropy and low complexity, indicating stochastic process characteristics. As the entropy *H* for maize is more significant than for the soybean, there is more genetic information for maize when we compare it with the soybean. Moreover, we also can say that the time series generated by corn has unpredictable patterns; that is, it has more random patterns for soybeans. It can be translated into a more blurred fractal pattern in the CGR of Fig. 2. On the other hand, soybean is more complex than maize, i.e., with higher complexity *C*. The statistical complexity quantifies the existence of non-trivial structures. In the cases of perfect order and total randomness, $C = 0$ means the data possesses no structure. Between these two extreme instances, an extensive range of possible values quantifies the level of structure in the data. The statistical complexity can detect subtle details of the dynamical processes that generate the data. In this sense, we can say that soybeans have a more complex structure than maize. This same result is corroborated by the CGR, where soybean has a more evident fractal structure than maize.

In the context of maize and soybean DNA sequences, it is critical to consider the C-value paradox, especially given the significant disparity in genome sizes between the two species. The “C-value paradox” is a term used in biology to describe the apparent disconnect between genome size and organism complexity^46,47^. Although maize has a much larger number of base pairs, this quantity does not translate into a more organized genomic structure and greater complexity of the organism.

One possible explanation is that the soybean genome may have a relatively lower proportion of repeated sequences and mobile genetic elements compared to corn, which contributes to a clearer organization and more uniform genomic structure. Furthermore, soybeans may have undergone processes that favored genome compaction and the elimination of unnecessary or redundant sequences, resulting in a more efficient and cohesive organization of DNA.

**Figure 6:**
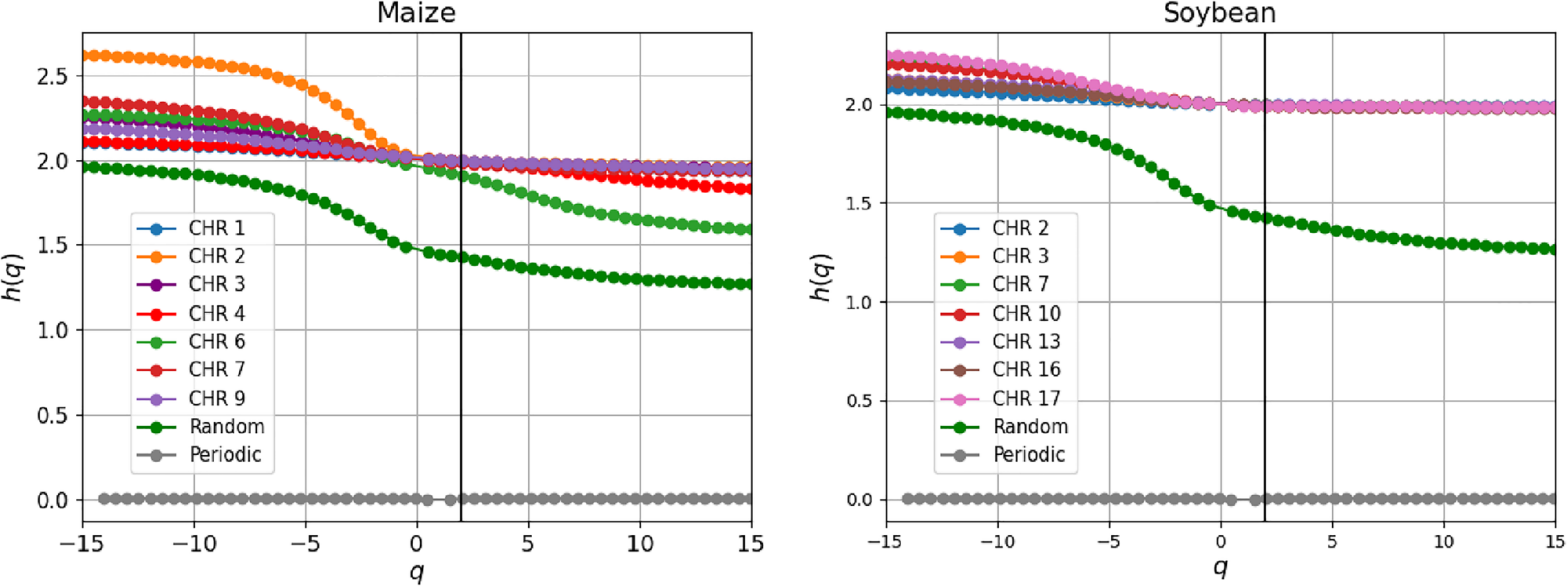
The Generalized Hurst exponents *h*(*q*) for maize (left) and soybean (right) chromosomes were chosen randomly. The vertical black line at $q = 2$ helps to visualize the values *h*(2). This same behavior is observed in the other chromosomes.

**Figure 7:**
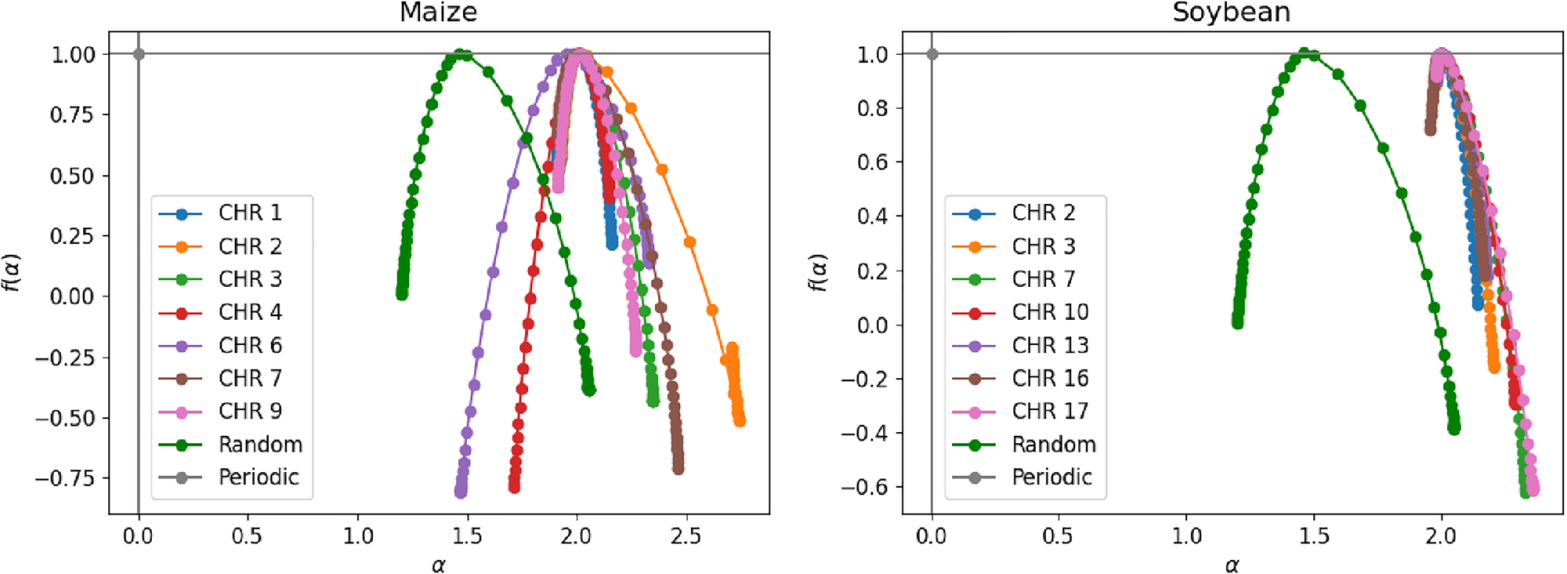
MF-DFA analysis of RNA sequences. The $f(\alpha )$ spectra vs scaling indices $\alpha$ for sequences of maize (left) and soybean (right) DNA.

### MF-DFA analysis

We also applied the MF-DFA analysis to all 30 chromosomes. We use a Python library for MF-DFA introduced in Ref.^48^ and available on Github^49^. We determine the generalized exponents and the multifractal spectra. We use the second-order polynomial fit (DFA2) over a segment interval *s* (100, 4, 000, 000) with step 1000 to obtain these results.

For comparison, we show two other artificial sequences: a periodic sequence constructed from the repetition of the letters “ATGC” 7, 500 times and another sequence with 30, 000 base pairs constituted of the letters “A”, “T”, “G”, and “C” randomly distributed. We made this comparison because these artificial time series present interesting behavior: The periodic series does not present a fractal pattern, and therefore, its fluctuation function is independent of q, while the random series presents a weak correlation between the nucleotides.

For the random sequence, one gets $H \lesssim 0.5$ and reveals a weakly correlated nucleotide sequence, as expected for a random sequence. For the periodic sequence, $h(q) = 0$ for all values of *q* (grey), and it reveals a non-fractal behavior. As seen in Fig. 6, for some chromosomal sequences, one obtains $0.97 \lesssim H$, indicating that fluctuations in base pair sequences exhibit a highly persistent nature. The other chromosomes present the same behavior, and the Hurst exponents’ values for each one are shown in the Table reftab:estatistica. Persistence is characterized by the tendency of the time series to be followed by positive values (long-range correlation) when presenting positive values in the sequence. It means that when one of the base pairs Adenine and Guanine occurs, and there is a tendency for these nitrogenous bases to continue appearing over a long period, the same behavior is valid for the non-occurrence of these bases.

The *h*(*q*) spectra for all chromosomal sequences show relatively small variation with *q*; see $\Delta h$ in Table 2. The width of the *h*(*q*) plot can give insights into the degree of multifractality in a time series. If the width is narrow, it suggests a weak correlation between different scales of the time series. It is a simple fractal structure that a small number of scaling factors can describe. On the other hand, a broad width indicates a strong correlation between different scales. On average, we got $\langle \Delta h\rangle _{maize}= 0.369$ for maize and $\langle \Delta h \rangle _{soybean}= 0.2915$ for soybean, indicating that maize has a more heterogeneous sequence than soybean, characterized by a well-defined multifractal structure with a long-range power-law correlation between nucleotides and a relatively more significant number of scale factors.

The multifractal spectra obtained from Eq. (16) for all the curves show concave behavior with maxima at scaling indices $\alpha = h(2)$. See Fig. 7 and Table 2. In the periodic sequence, the spectrum degenerates to a single point. The width of $f (\alpha )$ is a measure of the degree of multifractality: the greater the width, the more heterogeneous the fractal, i.e., the greater the complexity of the generating process of the analyzed series and the greater the difficulty in making predictions. On average we got $\langle \Delta \alpha \rangle _{maize}=0.495$ for maize and $\langle \Delta \alpha \rangle _{soybean}= 0.397$ for soybean. In this sense, maize has a greater mean variation, indicating that it has a more complex generator complex and is more difficult to make predictions about the time series.

Parameter *B* is more significant than 1 for most maize and soybean chromosomes. In this sense, we noticed that the soybean chromosomes present significant asymmetry. The left asymmetry indicates that the time series has higher complexity and variability at more minor scales, with fluctuations becoming less significant as the scale increases. On the other hand, chromosomes 4, 5 and 6, for maize, show right asymmetry and indicate that more significant fluctuations in chromosome sequences contribute more significantly to the multifractal spectrum.

The MF-DFA method is a powerful multifractal analysis tool and is a robust, well-known, widely used and easily applicable method. In addition to this, we can highlight other different analysis approaches that can be used to study vegetable sequences, such as multifractal detrended cross-correlation analysis, WTMM and its variants^50,51^. Different approaches can be used to address this problem and offer distinct and complementary perspectives on the (multi)fractal characteristics of plant genetic sequences. Combining and comparing these methods provides a more complete and robust understanding of the temporal dynamics of the systems studied, allowing deeper insights into their complexity and emergent behavior over time.

## Conclusion

We apply the Chaos game representation, ordinal patterns, and MF-DFA approach to study the characteristics of maize and soybean sequences. We investigated structural proprieties across multiple scales using these methods. The information obtained from this analysis helps classify and characterize genomic data. Through these approaches, it was demonstrated that:

- Through the Chaos Game Representation (CGR) method, we analyzed a set of DNA and protein sequences and generated fractal-like images that revealed unique patterns and features of the input sequences. The results from this method indicate that soybean sequences have a fractal structure more defined than maize sequences.
- This complexity in the soybean structure is also detected through the complexity measure *C*.
- CGR reveals the presence of power-law correlations at different scales for sequence DNA sequence. This result is corroborated by the Hurst exponent *H* values, in addition to indicating the persistent nature of the time series.
- Calculating the distance parameter *d* between all chromosomes, we conclude that the base pair sequences between the two species show high similarity.
- The mapping of base pairs of the sequences into numerical values informed us of the presence, in greater concentration, of the Adenine and Guanine bases in both species.
- The permutation entropy indicates that maize sequence is more random than soybean.
- Through the MF-DFA approach, we observe that, in the mean, the chromosomes from maize have a more complex multifractal structure than chromosomes from soybean; that is, more scaling factors are needed to characterize the sequence from maize than from soybean.
- The maize sequence presents a high degree of heterogeneity, characterized by the greater complexity of the time series’ generating process and complex prediction than the time series generated from soybean.
- The high left symmetry of the soybean sequences indicates that the time series has greater complexity and variability on small scales than the series generated by maize.
- The plane complexity-entropy reveals that both time series have stochastic process characteristics.
- In summary, maize sequences have a more complex and less random structure than maize. This complexity is translated through a better-defined fractal structure. Maize, on the other hand, has a more random and less complex structure.

Despite these important and promising results, we emphasize the need to connect these findings with biological meaning. The frequencies of k-mers may have implications for the occurrence of proteins in these vegetables. Furthermore, the MF-DFA analysis can have a lot to say about the mutations that these vegetables undergo over time. Therefore, a deeper approach that connects these results could be a promising next step.

Additionally, we stress the significance of conducting further research with more closely related species regarding phylogeny and genome size, as this is essential for extending and verifying the findings found thus far. These supplementary investigations will enable a deeper comprehension of the connections between genomic structures and provide context for the present findings. We aim to enhance our understanding of the fractal and complexity characteristics of the genomic sequences in these plants by integrating these supplementary investigations.

## Data Availability

The datasets analysed during the current study are available in the NCBI repository, https://www.ncbi.nlm.nih.gov/. All data analysed during this study are included in this published article and its supplementary information files.
